# Visualizing the Ensemble Structures of Protein Complexes Using Chemical Cross-Linking Coupled with Mass Spectrometry

**DOI:** 10.1007/s41048-015-0015-y

**Published:** 2015-12-28

**Authors:** Zhou Gong, Yue-He Ding, Xu Dong, Na Liu, E. Erquan Zhang, Meng-Qiu Dong, Chun Tang

**Affiliations:** CAS Key Laboratory of Magnetic Resonance in Biological Systems, State Key Laboratory of Magnetic Resonance and Atomic Molecular Physics, Wuhan Institute of Physics and Mathematics, Chinese Academy of Sciences, Wuhan, 430071 China; National Institute of Biological Sciences, Beijing, 102206 China

**Keywords:** Protein–protein interaction, Encounter complex, Fleeting complex, Ensemble refinement, Ambiguous distance restraint

## Abstract

**Graphical Abstract:**

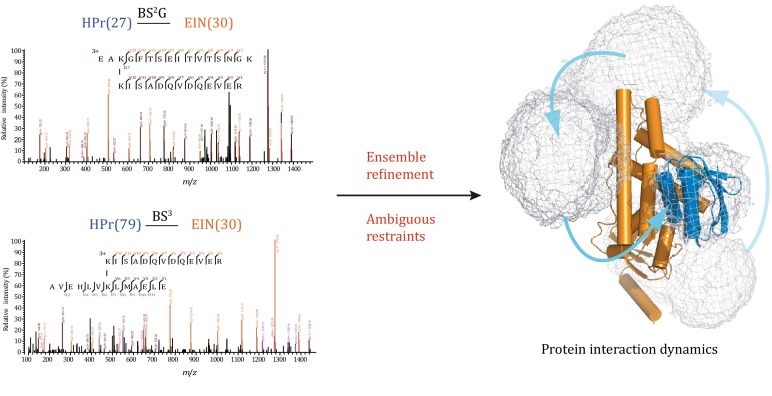

****Abstract**:**

Chemical cross-linking coupled with mass spectrometry (CXMS) identifies protein residues that are close in space, and has been increasingly used for modeling the structures of protein complexes. Here we show that a single structure is usually sufficient to account for the intermolecular cross-links identified for a stable complex with sub-µmol/L binding affinity. In contrast, we show that the distance between two cross-linked residues in the different subunits of a transient or fleeting complex may exceed the maximum length of the cross-linker used, and the cross-links cannot be fully accounted for with a unique complex structure. We further show that the seemingly incompatible cross-links identified with high confidence arise from alternative modes of protein-protein interactions. By converting the intermolecular cross-links to ambiguous distance restraints, we established a rigid-body simulated annealing refinement protocol to seek the minimum set of conformers collectively satisfying the CXMS data. Hence we demonstrate that CXMS allows the depiction of the ensemble structures of protein complexes and elucidates the interaction dynamics for transient and fleeting complexes.

**Electronic supplementary material:**

The online version of this article (doi:10.1007/s41048-015-0015-y) contains supplementary material, which is available to authorized users.

## **INTRODUCTION**

A protein interacts with other proteins to perform its function. The binding affinity or *K*_D_ value between two proteins ranges over ten orders of magnitude, and the resulting complex can be stable, transient or fleeting (Jones and Thornton 1996; Nooren and Thornton 2003). Examples of stable complexes include enzyme/enzyme inhibitor and antigen/antibody (Kastritis et al. 2011), while transient and fleeting complexes are often involved in cell signaling. Transient complexes are those with *K*_D_ values greater than 1 µmol/L, whereas fleeting complexes are three–four orders of magnitude weaker with *K*_D_ values in mmol/L (Vinogradova and Qin 2012; Xing et al. 2014; Liu et al. 2016).

Two transiently interacting proteins not only form a stereospecific complex, they can also form a series of nonspecific encounter complexes (Tang et al. 2006; Fawzi et al. 2010; Schilder and Ubbink 2013). Encounter complexes are important structural intermediates, and facilitate the formation of the stereospecific complex. Yet, encounter complexes constitute only a minor population of the total complex, and are difficult to study (Berg et al. 1981; Schreiber and Fersht 1996; Gabdoulline and Wade 2002). With the *K*_D_ value in mmol/L, the distinction between specific and non-specific complexes starts to blur, and the subunits in a fleeting complex often adopt a variety of conformations (Tang et al. 2008; Liu et al. 2012). As such, to characterize the structure of a protein complex, especially a transient or fleeting complex, it often requires an ensemble description to recapitulate the different conformational states.

Chemical cross-linking of proteins coupled with mass spectrometry analysis (CXMS) is an emerging technique to investigate protein-protein interactions (Rappsilber 2011; Herzog et al. 2012; Kalisman et al. 2012; Lasker et al. 2012; Walzthoeni et al. 2013; Politis et al. 2014). Amine-specific homo-bifunctional cross-linkers, including bis-sulfosuccinimidyl suberate (BS^3^) and bis-sulfosuccinimidyl glutarate (BS^2^G), are commonly used. Recently, carboxylate-specific cross-linkers reactive towards glutamate or aspartate residues, such as pimelic acid dihydrazide (PDH; Leitner et al. 2014), were added to the CXMS toolbox. In theory, two primary amine groups (either lysine side chain or protein N-terminus) or two carboxylate groups (either glutamate or aspartate side chains) that are close in space can be covalently linked. The cross-linked residues can be identified with the use of a database search engine (Rinner et al. 2008; Yang et al. 2012), and each intermolecular cross-link can be converted to a distance restraint for modeling the complex structure (Rappsilber 2011; Kalisman et al. 2012; Walzthoeni et al. 2013; Schmidt and Robinson 2014).

As CXMS has been increasingly used for the structural characterization of protein complexes, two technical issues have become apparent (Rappsilber 2011; Merkley et al. 2014). First, only a fraction of the cross-links expected from the known structure of a protein complex are experimentally observed. This can be due to low accessibility and reactivity of the involved residues (Leitner et al. 2014). Second and more intriguingly, for a subset of cross-links, the theoretical distance between two cross-linked residues, as calculated from the specific complex structure, sometimes exceeds the maximum length of the cross-linker (Kahraman et al. 2013). Incorrect identification of cross-linked peptides has been blamed for such discrepancies (Zheng et al. 2011; Kalisman et al. 2012). Yet, with the most stringent criteria that essentially eliminate false identifications, sometimes there remain cross-links violating the distance limits (Lossl et al. 2014). So what are the origins of these “incompatible” cross-links?

CXMS data have been recently implemented in ROSETTA software package for modeling protein complex structures (Kahraman et al. 2013; Lossl et al. 2014). The approach aims to obtain a single structure that satisfies CXMS restraints and has the lowest ROSETTA energy score, and is suited for characterizing stable complex structures. Nevertheless, as transient and fleeting complexes can adopt a multitude of conformational states, a single-conformation representation may not suffice. Here we show that the highly reliable but seemingly incompatible cross-links arise from alternative modes of protein–protein interactions. We present a rigid-body refinement protocol against all the experimental cross-links, and show that an ensemble representation comprising multiple conformers of the complex is often required when characterizing transient and fleeting complexes.

## **RESULTS**

### **Refinement of the stable complex structure**

To refine against intermolecular CXMS restraints, we treated each subunit as a rigid body. Any two cross-linked lysine residues were restrained to have their C_α_-C_α_ distance to be less than the maximum length of the corresponding cross-linker using a square-well pseudo-energy potential. BS^3^ and BS^2^G covalently link lysine residues <24 Å and <20 Å apart, respectively, as measured from C_α_ to C_α_ atoms (Lee 2009; Kahraman et al. 2011). Cross-links may also involve protein N-terminus; when fully extended, the maximum C_α_-C_α_ distance between an N-terminal residue and a lysine is 15 Å for BS^2^G and 19 Å for BS^3^.

**Fig. 1:**
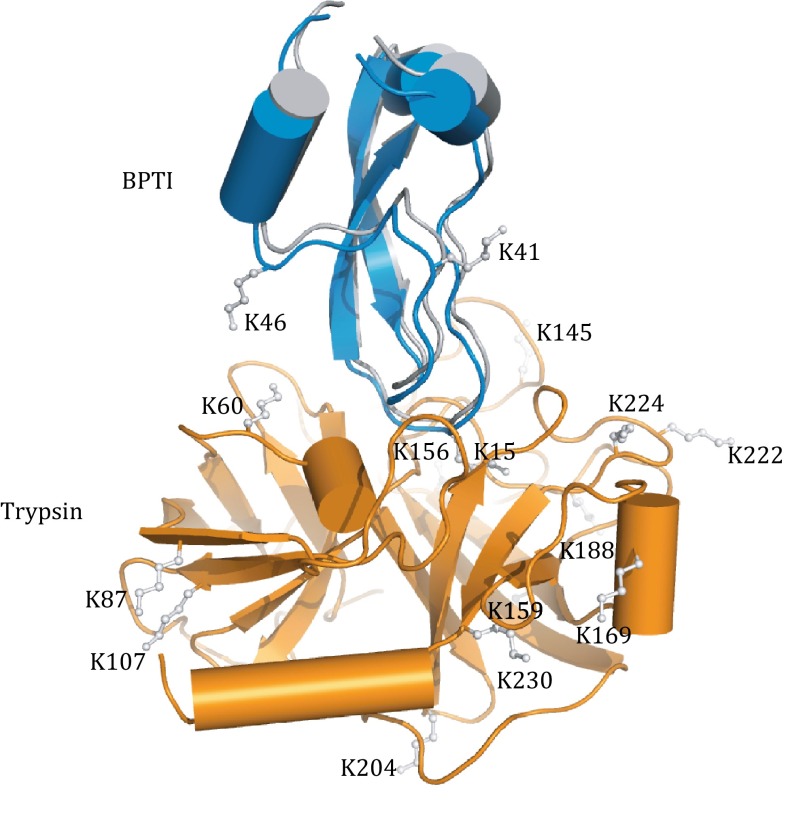
Comparison between the CXMS model and the X-ray structure for the complex between trypsin and BPTI. The two structures are superimposed by trypsin (*orange cartoon*), and BPTI in the CXMS model and in the crystal structure (PDB code 2PTC) are colored *gray* and *blue*, respectively. The CXMS model was obtained by refining against 17 theoretical inter-molecular cross-links. The RMS difference of backbone heavy atoms between the two complex structures is 0.54 Å. Lysine residues involved are *labeled*

We then assessed the refinement protocol on the complex between trypsin and bovine pancreatic trypsin inhibitor (BPTI), a stable complex with a *K*_D_ value of ~60 fmol/L (Marquart et al. 1983; Kastritis et al. 2011). Based on the known structure of the complex (PDB code 2PTC), there can be a maximum of 17 theoretical inter-subunit lysine-lysine cross-links with BS^3^ cross-linking reagent (Table S1). Starting from the structures for the free proteins (PDB codes 4GUX and 1JV8, for trypsin and BPTI, respectively), we fixed the coordinates of trypsin and allowed BPTI to freely rotate and translate as a rigid body. With simulated annealing, we refined the complex structure against the CXMS restraints, with additional van der Waals repulsive term employed. Calculating one structure takes less than 2 min on a single core of Intel Xenon 5620 CPU. Repeating the calculation from different starting positions for the two subunits afforded a set of highly converged structures with overall root-mean-square deviation (RMSD) for backbone heavy atoms almost 0 Å. Importantly, the RMS difference between the CXMS model and the crystal structure was only 0.54 Å (Fig. 1).

### **Further assessment of the rigid-body refinement protocol**

**Fig. 2:**
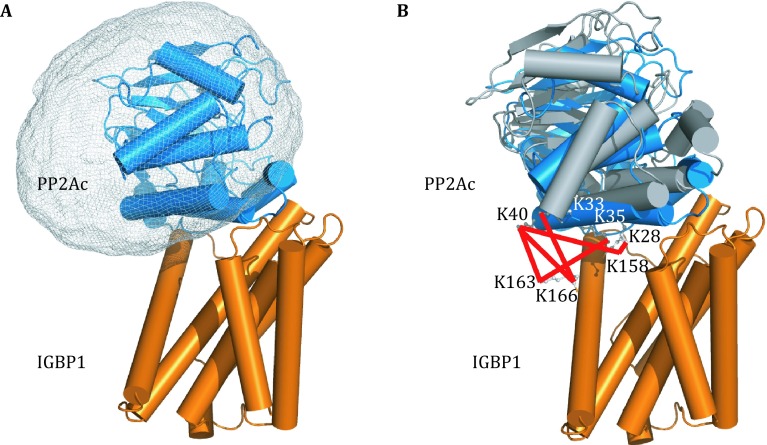
CXMS model obtained for the complex between PP2Ac and IGBP1. **A** The distribution of PP2Ac with respect to IGBP1 (*orange cartoon*) is shown as atomic probability map, plotted at 30% threshold and shown as *gray meshes*. **B** The RMS difference between the CXMS model (*gray cartoon* for PP2Ac) and the crystal structure of the complex (PDB code 4IYP) can be as small as 2.8 Å. With PP2Ac superimposed, IGBP1 in the crystal structure is shown as *blue cartoon*. Cross-linked lysine residues are *labeled* and the intermolecular cross-links are shown as *red* *lines*

In practice, however, it is rare to have as many as 17 intermolecular cross-links for a complex with the size of trypsin/BPTI (281 residues total and 18 lysine residues). Often, only a few cross-links can be experientially identified. To assess how robust the refinement protocol is with fewer CXMS restraints, we obtained CXMS data from the published studies (Herzog et al. 2012; Kahraman et al. 2013) for the complex between protein phosphatase 2A catalytic subunit (PP2Ac) and immunoglobulin binding protein 1 (IGBP1). PP2Ac and IGBP1 interact with each other with a *K*_D_ value of ~300 nmol/L (Jiang et al. 2013), and six intermolecular cross-links were identified between Lys^28^-Lys^158^, Lys^33^-Lys^166^, Lys^35^-Lys^163^, Lys^40^-Lys^158^, Lys^40^-Lys^163^, and Lys^40^-Lys^166^ (from PP2Ac to IGBP1) (Herzog et al. 2012). Starting from the structures for free PP2Ac (PDB code 2NYL) and IGBP1 (PDB code 3QC1) proteins, we obtained their complex structures by refining against the CXMS distance restraints. The probabilistic distribution was computed for PP2Ac with respect to IGBP1 in all the structural models and was shown as atomic probability map (Schwieters and Clore 2002), which encompassed the known complex structure (Fig. 2A). Importantly, the overall backbone RMS difference between the CXMS models and the crystal structure for PP2Ac/IGBP1 complex was as small as 2.8 Å (Fig. 2B) (Jiang et al. 2013).

Then what is the minimum number of intermolecular cross-links needed to model the complex structure? With the use of three experimental cross-links involving PP2Ac Lys^40^ (Lys^40^-Lys^158^, Lys^40^-Lys^163^, and Lys^40^-Lys^166^), the resulting structures took up similar positions (Fig. S1A) as the structures calculated using the full set of CXMS restraints, though a bit more scattered. With only one CXMS restraint, for example from PP2Ac Lys^35^ to IGBP1 Lys^163^, the modeling still afforded a set of CXMS models that are similar to those calculated with the full set of experimental CXMS restraints (Fig. S1B). Thus, the more CXMS restraints were incorporated, the more converged the resulting models were. We also performed the structural refinement using five out of the six cross-links, and then back-calculated the C_α_-C_α_ distance for the unused cross-link. Except for the cross-link between PP2Ac Lys^28^ and IGBP1 Lys^158^, the calculated distances are mostly within the maximum length stipulated by the corresponding cross-linker (Table S2). Thus, the cross-link between PP2Ac Lys^28^ and IGBP1 Lys^158^ afforded a key restraint about the complex structure, and owing to the sparsity of the inter-molecular cross-links, this cross-link is not redundantly provided by other cross-links.

Using CXMS, we characterized the complex between CDK9 and Cyclin-T1. This complex is responsible for transcription elongation, and its two subunits interact with each other at a *K*_D_ value of ~300 nmol/L (Baumli et al. 2008). We focused our attention on the intermolecular cross-links that were identified twice or more, for which the probability of being observed by random chance was below 10^−8^ for at least one instance and below 10^−3^ for additional instances (a false discovery rate cutoff of 0.05, an *E*-value cutoff rate of 10^−3^, spectral count ≥2, and the best *E*-value cutoff of 10^−8^). With these stringent criteria, it would be unlikely that the cross-links were identified by random chance, and the remaining cross-links should be correctly assigned. Three intermolecular cross-links were identified for CDK9/Cyclin-T1 (Table 1) and the corresponding MS2 spectra are shown in Fig. S2. For each, the two linked lysine residues were found within the maximum length of the cross-linker, as calculated from the known structure of the complex (Baumli et al. 2008).

**Table 1:** Intermolecular cross-links observed for transient and fleeting protein complexes

Cross-linked pairs	BS^2^G	BS^3^	Total spectra	Best *E*-value^a^	C_α_-C_α_ (Å)^b^	Remarks^c^
Cyclin-T1(6)–CDK9(144)	13	15	28	1.7 × 10^−10^	18.5	–
Cyclin-T1(6)–CDK9(74)	9	35	44	3.7 × 10^−11^	11.2	–
Cyclin-T1(100)–CDK9(56)	30	25	55	2.5 × 10^−13^	9.3	–
EIN(1)–HPr(24)^d^	26	0	26	2.4 × 10^−18^	49.1	EC-III
EIN(1)–HPr(49)	23	14	37	1.3 × 10^−32^	45	EC-III
EIN(29)–HPr(1)^e^	0	18	18	9.4 × 10^−16^	49.1	EC-II
EIN(30)–HPr(1)^e^	0	7	7	1.1 × 10^−18^	46.9	EC-II
EIN(30)–HPr(24)	25	74	99	2.1 × 10^−24^	36.8	EC-II
EIN(30)–HPr(27)	31	12	43	1.2 × 10^−23^	35.3	EC-II
EIN(30)–HPr(49)	3	1	4	7.8 × 10^−42^	29.9	EC-II
EIN(30)–HPr(79)^e^	0	10	10	2.2 × 10^−10^	49.5	EC-II
EIN(49)–HPr(24)^d^	15	0	15	8.4 × 10^−21^	22.3	EC-I
EIN(49)–HPr(49)^e^	0	36	36	2.0 × 10^−25^	26.5	EC-I
EIN(49)–HPr(72)^d^	8	0	8	1.3 × 10^−18^	35.2	EC-I
EIN(58)–HPr(24)	1	13	14	2.5 × 10^−26^	15.4	SC
EIN(238)–HPr(24)^d^	9	0	9	2.5 × 10^−18^	56.1	EC-III
Ub(6)–Ub(48)	24	24	48	5.1 × 10^−17^	–	–
Ub(6)–Ub(63)	23	2	25	4.5 × 10^−17^	–	–
Ub(11)–Ub(48)	3	83	86	1.6 × 10^−24^	–	–
Ub(29)–Ub(48)	15	22	37	1.8 × 10^−12^	–	–
Ub(33)–Ub(48)	174	78	95	7.5 × 10^−24^	–	–
Ub(48)–Ub(48)	67	103	170	3.5 × 10^−18^	–	–
Ub(63)–Ub(63)	8	0	8	3.3 × 10^−19^	–	–

^a^The best *E*-value among all the MS2 spectra for each cross-link. *E*-value is the probability of observing the cross-link by chance

^b^Distance was calculated from the known stereospecific complex structure, PDB accession code 3EZA. No uniquely defined structure is available for the ubiquitin dimer

^c^Designations of the conformational clusters for EIN–HPr complexes

^d^Observed with BS^2^G, but not with BS^3^

^e^Observed with BS^3^, but not with BS^2^G

**Fig. 3:**
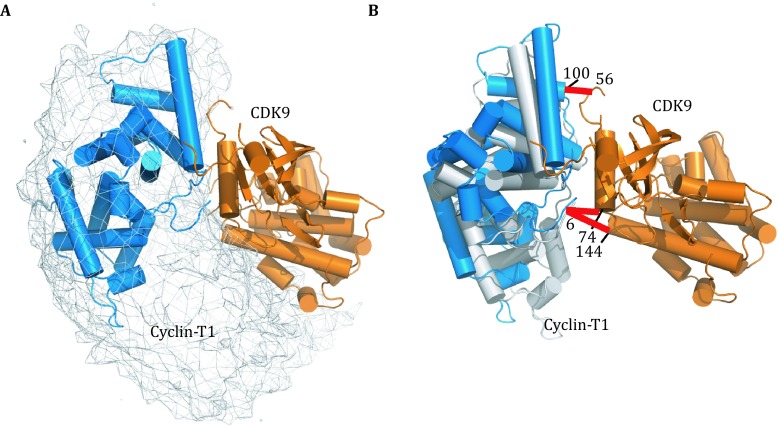
Structural model for the CDK9/Cyclin-T1 complex refined against intermolecular CXMS restraints. **A** The distribution of Cyclin-T1 with respect to CDK9 (*orange cartoon*) is represented as an atomic probability map plotted at a 10% threshold (*gray mesh*). **B** A selected CXMS model, shown as *orange* and *gray cartoon* for CDK9 and Cyclin-T1, respectively. For comparison, the CDK9 of the crystal structure (PDB code 3BLH) is superimposed, and the Cyclin-T1 crystal structure is shown as a *blue cartoon*. The root-mean-square deviation between the two complex structures is 2.86 Å. Each set of two cross-linked residues is denoted with a *red bar*

We treated each subunit in CDK9/Cyclin-T1 as a rigid body, and refined against the intermolecular CXMS distance restraints: two cross-linked lysine residues were restrained to have their C_α_-C_α_ distance to be less than the maximum length of the corresponding cross-linker using a square-well energy potential. Since each intermolecular cross-link was observed with both BS^2^G and BS^3^ cross-linkers (Table 1), we restrained the C_α_-C_α_ distance to be shorter than the length of BS^2^G (20 Å for lysine-lysine cross-links and 15 Å for lysine-protein N terminus cross-links). In the refinement, the coordinates for one subunit, CDK9, were fixed, while the other subunit, Cyclin-T1, was grouped as a rigid body, given full translational and rotational freedoms. A single intermolecular CXMS restraint was readily satisfied, but the resulting complex model was poorly converged, with Cyclin-T1 dangling along one side of CDK9 (Fig. S3). As Lys^74^ and Lys^144^ are adjacent to each other in CDK9, cross-links of Cyclin-T1 Lys^6^ to these two residues provided redundant information about the complex structure. Cyclin-T1 Lys^100^ and CDK9 Lys^56^ are located at the other side of the complex; as a result, the refinement against the corresponding cross-link restraint afforded a different but overlapping distribution of the complex. With all three restraints used, a narrower distribution was obtained (Fig. 3A). Significantly, the structural models based on CXMS restraints encompassed the known crystal structure of CDK9/Cyclin-T1, and the pairwise RMS difference between the CXMS model and the PDB structure was as small as 2.86 Å (Fig. 3B). Thus, we show that the CDK9/Cyclin-T1 complex can be modeled as a single conformer, based on sparse CXMS distance restraints.

### **CXMS analyses of transient and fleeting complexes**

We then performed CXMS analysis for EIN/HPr and ubiquitin homodimeric complexes using BS^2^G and BS^3^. EIN and HPr are involved in signal transduction for bacterial sugar uptake and interact with each other with a *K*_D_ value of ~7 µmol/L (Suh et al. 2007). Ubiquitin is an important signaling protein in cell and can noncovalently dimerize with a *K*_D_ value of ~5 mmol/L (Liu et al. 2012). Using the same stringent criteria described above, intermolecular cross-links for the two complexes are also presented in Table 1, and the corresponding MS2 spectra are shown in Figs. S4 and S5. A total of 13 intermolecular cross-links were identified for EIN/HPr, but only one of them (EIN Lys^58^ to HPr Lys^24^) was found consistent with the stereospecific complex structure (Garrett et al. 1999). For validation, we also performed CXMS analysis for EIN/HPr using PDH (Leitner et al. 2014) as the cross-linking reagent.

In order to identify intermolecular cross-links between two ubiquitin subunits in a ubiquitin homodimer, we performed CXMS analysis on a mixture of ^14^N-labeled (natural isotope abundance) and ^15^N-labeled ubiquitin proteins (Liu et al. 2012). The cross-links between ^14^N- and ^15^N-labeled peptides with characteristic MS1 spectra (Fig. S6) should only arise from intermolecular interactions (Taverner et al. 2002). In this way, we identified a total of seven intermolecular cross-links for the ubiquitin homodimer.

### **Ensemble structure refinement of protein encounter complexes**

**Fig. 4:**
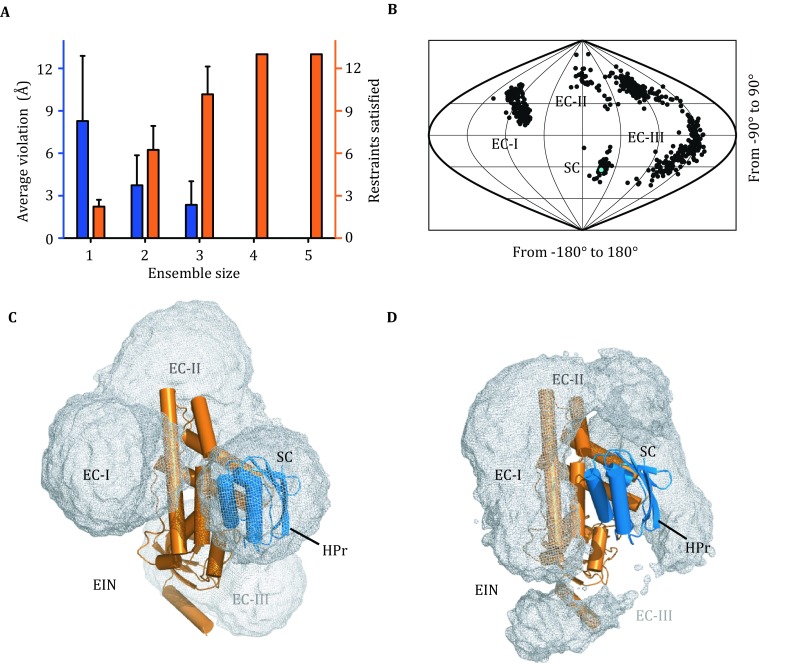
Ensemble refinement for the complex structure between EIN and HPr. **A** Average violation of CXMS distance restraint (*blue axis* on the *left*) and the number of the satisfied restraints (*orange axis* on the *right*) versus the number of conformers representing the complex. With four or more conformers, all CXMS restraints can be satisfied. **B** *Spherical coordinates* for the four-conformer ensemble structures showing the distribution of HPr with respect to EIN. In each ensemble structure, the HPr is found in four clusters, namely EC-I, EC-II, EC-III, and SC. For comparison, the structure for EIN/HPr stereospecific complex (PDB code 3EZA) is indicated as a *cyan dot*. **C** Atomic probability map of the distribution of HPr with respect to EIN in the ensemble structure refined against intermolecular CXMS restraints. The difference clusters of CXMS conformers are *labeled*. **D** Atomic probability map of the distribution of HPr with respect to EIN in the ensemble structure refined against intermolecular PRE data. The NMR ensemble was calculated based on the previously published data (Tang et al. 2006). EIN is fixed and shown as *orange cartoon*, the distribution of HPr is shown as *gray meshes* and plotted at 20% threshold. For comparison, the stereospecific complex structure is superimposed, with HPr shown as *blue cartoon*, and the four clusters are also marked

To account for the experimental cross-links and to model the structure of EIN/HPr complex, we fixed the position of EIN and treated HPr as a rigid body given rotational and translational freedoms. The intermolecular cross-links could not be satisfied with a single-conformer representation of the complex, as the restraints were consistently violated with an average violation >8 Å (Fig. 4A). This means that in addition to the stereospecific complex, HPr sampled a multitude of conformations with respect to EIN, which were captured by cross-linking. Thus, we invoked ensemble representation for the complex—with EIN fixed, HPr was represented as multiple conformers. We treated each intermolecular cross-link as an ambiguous restraint (Nilges 1995), and defined the CXMS energy averaged over all the conformers in the ensemble with a steep dependence on the C_α_-C_α_ distance. In this way, a CXMS restraint could be satisfied providing that it was accounted for by at least one conformer in the ensemble. The ensemble refinement showed that a minimum of four conformers was required to fully satisfy the intermolecular CXMS restraints with an average distance violation close to 0 Å (Fig. 4A). Too large an ensemble size, however, would lead to over-fitting. When using five conformers to represent the complex, HPr in the additional conformers were found scattering around, making no contribution to the CXMS energy (Fig. S7).

Using a spherical coordinate system, we projected the positions of HPr with respect to EIN in the CXMS models to lower dimensions. In the 2D plot, HPr was found in four distinct clusters (Fig. 4B), thus explaining the requirement of four conformers in the ensemble. One cluster (SC) contained conformers overlapping with the known complex structure, and therefore accounted for the stereospecific EIN/HPr interactions. HPr was positioned away from the specific interface with EIN in the other three clusters (EC-I, EC-II and EC-III), which represented non-specific interactions between EIN and HPr. Each cluster of conformers accounted for multiple intermolecular cross-links (Table 1).

We could cross-validate the ensemble structure modeled from lysine-lysine cross-links with the CXMS restraints from a different cross-linking reagent, PDH (Leitner et al. 2014). For a pair of PDH cross-linked glutamate residues, the C_α_-C_α_ distance should be less than 22 Å. With high confidence, the PDH cross-links were identified between EIN Glu^41^ and HPr Glu^85^ and between EIN Glu^67^ and HPr Glu^85^ (Fig. S8). Calculated from the stereospecific complex structure (Garrett et al. 1999), the C_α_-C_α_ distances for these two pairs of residues were 41.2 and 12.9 Å, respectively. Clearly, the cross-link between EIN Glu^41^ and HPr Glu^85^ could not be accounted for with the stereospecific complex structure alone. In the four-conformer ensemble structure modeled from BS^2^G/BS^3^ CXMS data, however, the averaged C_α_-C_α_ distance between EIN Glu^41^ and HPr Glu^85^ was 23.1 ± 4.9 Å.

Previously, EIN/HPr complex has been characterized with paramagnetic nuclear magnetic resonance (NMR), and it was shown that EIN and HPr form a multitude of encounter complexes, which facilitate the formation of the stereospecific complex (Tang et al. 2006; Fawzi et al. 2010). Protein encounter complexes are of low occupancies and short lifetimes. Previous NMR studies estimated that encounter complexes made up less than 10% of the total EIN/HPr complex, thus putting the apparent *K*_D_ value for the encounter interactions >10 mmol/L (Fawzi et al. 2010). Importantly, the distribution of HPr relative to EIN modeled on the basis of CXMS data (Fig. 4C) resembles the EIN/HPr encounter complexes previously depicted using NMR spectroscopy (Fig. 4D).

### **Ensemble structure refinement of a fleeting complex**

Performing CXMS experiments on an equimolar mixture of ^15^N- and ^14^N-labeled ubiquitin proteins, we identified five inter-molecular cross-links. We fixed the coordinates for one ubiquitin, and allowed the other one to move. A single conformation for the ubiquitin dimer failed to satisfy all the restraints, with average violations ~2 Å. Hence we represented the ubiquitin dimer with two, three, and four conformers, with *C*_2_ non-crystallographic symmetry enforced for each pair of ubiquitin dimer. The CXMS restraints could be satisfied with an *N* = 2 ensemble. Increasing the size of the ensemble did not improve the agreement between experimental and calculated C_α_-C_α_ distances, and the additional conformers in the *N* = 3 and 4 ensemble scattered around with respect to its dimer partner (Fig. S9). Thus, the *N* = 2 ensemble was sufficient to describe the dynamic interactions between two ubiquitin proteins.

**Fig. 5:**
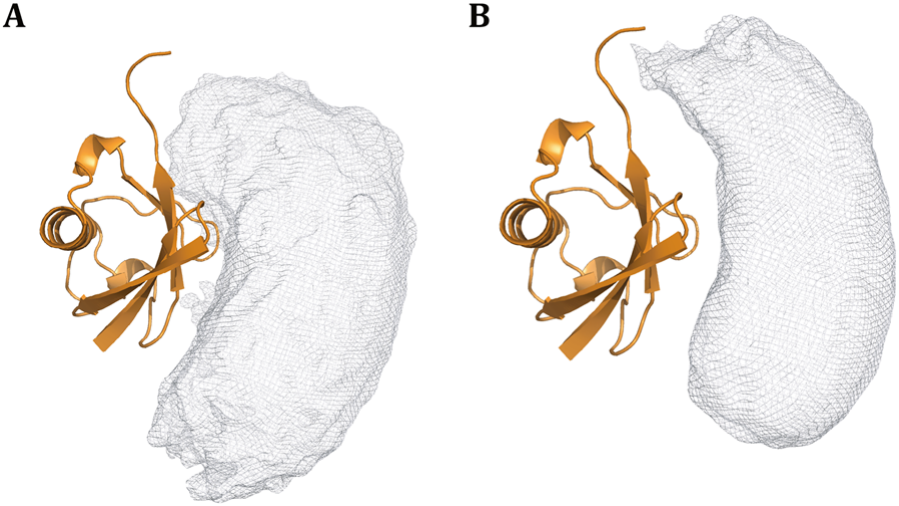
Ensemble structure for the ubiquitin homodimer. With one ubiquitin subunit fixed (*orange cartoon*), the probabilistic distribution of the other ubiquitin subunit in the dimer is plotted at 20% threshold (*gray meshes*). The ensemble structures of ubiquitin homodimer were calculated by refining against **A** intermolecular CXMS restraints or **B** intermolecular NMR restraints

In the CXMS models, the two ubiquitins adopt a variety of orientations (Fig. 5A), characteristic of fleeting protein-protein interactions (Liu et al. 2016). This also explains why Lys^48^ in one ubiquitin was able to cross-link to five different lysine residues, except for Lys^27^ and Lys^63^, in the other ubiquitin. Importantly, the two subunits interacted at the β-sheet region in the CXMS models, and the distribution of the CXMS models was in good agreement with a previous NMR characterization of the ubiquitin homodimer (Fig. 5B).

## **DISCUSSION**

CXMS has been increasingly used to characterize protein-protein interactions and to model protein complex structures (Walzthoeni et al. 2013; Schmidt and Robinson 2014). However, when experimental cross-links cannot be accounted for with a unique structure, previous CXMS applications generally ignored “incompatible” ones or relaxed the C_α_-C_α_ distance restraints (Herzog et al. 2012; Politis et al. 2014). Here we show that CXMS is exquisitely sensitive to encounter and fleeting protein-protein interactions that have apparent *K*_D_ values in mmol/L, and those seemingly incompatible cross-links contain the information about the dynamics of protein-protein interactions.

To account for the intermolecular cross-links identified with high confidence, we established a rigid-body refinement protocol. The protocol enabled the depiction of the relative subunit distributions in a complex. We first show that the refinement protocol can model the structures of stable complexes to high precision and accuracy. For transient and fleeting ones, however, when a single conformation failed to satisfy all the intermolecular cross-links, we invoked ambiguous distance restraints, in which a distance restraint was accounted for by any one of the conformers in the ensemble (Fig. S10). Demonstrated with EIN/HPr and ubiquitin homodimeric complexes, we showed that the resulting structures satisfied the experimental intermolecular cross-links and recapitulated alternative modes of protein-protein interactions. Moreover, the lysine- and carboxylate-specific cross-links for the EIN/HPr complex corroborate each other, which attests the power of CXMS in revealing the dynamics in protein interactions. Nevertheless, it should be noted that, though a qualitative validation of the ensemble structure can be readily performed, a complete cross-validation may not be feasible owing to the sparsity of the CXMS restraints.

Protein interaction dynamics have been mostly characterized using NMR spectroscopy. Though NMR afforded more structural details than CXMS does, it only works for relatively small protein complexes and requires a large amount of isotopically labeled proteins. In contrast, CXMS is not limited by the size of the proteins, and can be performed on µg or ng of proteins of natural isotope abundance. CXMS is often used conjunction with other techniques like electron microscopy (EM; Rappsilber 2011; Thalassinos et al. 2013). Nevertheless, the data from other technique are sometimes at odds with the CXMS data (Plaschka et al. 2015). Since proteins dynamically interact with each other, we envision that the ensemble refinement protocol presented herein will allow the reconciliation of different types of data and enable the characterization of subunit rearrangement in these large complexes. The method described herein does not take into account the flexibility of each subunit. Yet we anticipate that CXMS would allow the visualization of the dynamics for each individual protein, providing that a large number of intra-molecular cross-links of high confidence are identified using cross-linking reagents of different lengths and chemical properties.

## **MATERIALS AND METHODS**

### **Cross-linking reaction and analysis**

CDK9, Cyclin-T1, EIN, HPr, and ubiquitin proteins were purified as previously described (Garrett et al. 1999; Baumli et al. 2008; Liu et al. 2012). To prepare ^15^N-labeled protein, bacterial cells expressing ubiquitin were grown in M9 minimum medium with U-^15^NH_4_Cl as the sole nitrogen source. The two subunits in each complex were mixed at a 1:1 ratio—0.6 µmol/L for CDK9/Cyclin-T1, 16 µmol/L for EIN/HPr and 70 µmol/L for the ubiquitin homodimer. Cross-linking reactions were performed at room temperature in 20 mmol/L HEPES buffer (pH 8.0, 7.2 and 7.5 for CDK9/Cyclin-T1, EIN/HPr and ubiquitin, respectively) containing 150 mmol/L NaCl and 0.5 mmol/L BS^3^ (Thermo Scientific) or BS^2^G (Thermo Scientific) for 1 h, and were quenched with 20 mmol/L NH_4_HCO_3_. Cross-linking reactions using PDH for EIN/HPr complex were performed at 37 °C in 20 mmol/L HEPES buffer pH 7.2 containing 150 mmol/L NaCl and 11 mmol/L 4-(4,6-dimethoxy-1,3,5-triazin-2-yl)-4-methylmorpholinium chloride for 1 h, and were quenched with 20 mmol/L NH_4_HCO_3_. The proteins were subsequently precipitated with ice-cold acetone, air dried, and resuspended in 8 mol/L urea, 100 mmol/L Tris pH 8.5. The cross-linked samples were assessed with SDS-PAGE; about 30%–50% of the protein remains monomeric, whereas the remaining proteins correspond to the singly cross-linked form.

After trypsin (Promega) digestion, LC-MS/MS analysis was performed on an Easy-nLC 1000 UPLC (Thermo Fisher Scientific) coupled with a Q Exactive Orbitrap mass spectrometer (Thermo Fisher Scientific). The top ten most intense precursor ions from each full scan (resolution 70,000) were isolated for MS2 analysis. The pLink (Yang et al. 2012) program was used to search a database containing the sequences of the proteins in question and the cross-linked peptides were identified with the following criteria: false discovery rate smaller than 0.05 followed by an *E*-value cutoff of 10^−3^ at the spectral level; at the peptide level, spectral count ≥2 and the best *E*-value <10^−8^ for each identification. The lower the *E*-value, the less likely the putative identification is a false discovery (Yang et al. 2012). For each complex, the cross-linking reaction was repeated twice on different samples, which afforded almost identical cross-links.

To identify the intermolecular cross-links between two ubiquitin molecules, we mixed the ^15^N- and ^14^N-labeled (natural isotope abundance) ubiquitin at a 1:1 ratio. The ^14^N-/^14^N-labeled and ^15^N-/^15^N-labeled cross-linked peptide pairs were identified using pLink (Yang et al. 2012). Based on a strategy previously described (Taverner et al. 2002; Petrotchenko et al. 2014), we assigned cross-links between the ^15^N and the ^14^N-labeled peptides as intermolecular if the ratio in mass intensity in liquid chromatography of ^15^N-/^14^N-labeled (or ^14^N-/^15^N-labeled) cross-linked peptide relative to the corresponding ^14^N-/^14^N-labeled (or ^15^N-/^15^N-labeled) cross-linked peptide in the extracted ion chromatogram is >0.14. At this ratio, the intermolecular contribution is >25%.

### **Refinement of protein complex structures**

The starting structures for the specific complexes and for constituting proteins were retrieved from the PDB. The accession codes for trypsin, BPTI, and trypsin/BPTI complex are 4GUX, 1JV8, and 2PTC, respectively. The accession codes for PP2Ac and PP2Ac/IGBP1 complex are 2NYL and 4IYP (Jiang et al. 2013), respectively. Only the coordinates for the catalytic core domain were extracted from the PDB structure 2NYL. The coordinates for IGBP1 in the complex were obtained from the PDB structure 3QC1 (free) and 4IYP (bound to PP2Ac). Since many residues in free IGBP1 structure are missing (residues V122–M144), the free structure was spliced with the bound structure, and the resulting structure was solvated in a cubic box containing the TIP3P water molecules with a 10 Å padding in all directions. The structure was subjected 10 ns MD simulation in Amber 14 (Case et al. 2012) to relax the conformation, to generate the initial coordinates for the unbound IGBP1. The accession code for the CDK9/Cyclin-T1 complex was 3BLH. The accession codes for EIN, HPr, and EIN/HPr complexes were 1ZYM, 1POH, and 3EZA (Garrett et al. 1999), respectively. The PDB accession code for ubiquitin monomer is 1UBQ (Vijay-Kumar et al. 1987). The theoretical CXMS distance restraints for trypsin/BPTI were calculated using Xwalk (Kahraman et al. 2011) with 24 Å cutoff. The intermolecular cross-links for PP2Ac/IGBP1 complex were taken from a previous study (Herzog et al. 2012). In that report, the authors identified seven cross-links, one of which involves IGBP1 Lys^306^; since the known structure for IGBP1 encompasses residues 1–221, this cross-link is not used for the structural refinement.

Structural refinement against the CXMS restraints was performed using Xplor-NIH (Schwieters et al. 2006). The refinement started from the coordinates for the free proteins. Each protein subunit was treated as a rigid body, and only CXMS and van der Waals repulsive terms between the subunits are considered. In the refinement, one subunit was fixed, and the other subunit was manipulated with a random rotation and translation, away from the fixed subunit. For each intermolecular cross-link, a square-well energy function was used to enforce the C_α_-C_α_ distance of the cross-linked lysine residues less than 24 and 20 Å for the BS^3^ and BS^2^G cross-links, respectively (Lee 2009; Kahraman et al. 2011). The upper limits of the distance restraints for cross-linking involving a protein N-terminus were 19 and 15 Å for the BS^3^ and BS^2^G cross-linkers, respectively. The lengths correspond to a fully extended cross-linker and side chains of two cross-linked residues; no energy penalty was applied when the back-calculated C_α_-C_α_ distance was within the maximally allowed lengths. The penalty for a distance violation was defined as *k*Δ^2^, as the force constant *k* was gradually ramped from 1 to 30 kcal/(mol · Å^2^), as the bath temperature cooled from 3000 K to room temperature in the simulated annealing protocol. Upper limits for BS^2^G were used when intermolecular cross-links were observed with both BS^2^G and BS^3^; upper limits for BS^3^ were used for intermolecular cross-links were observed with only BS^3^. In addition to the distance restraint derived from CXMS, the restraints also included covalent terms, and van der Waals repulsive energy term. For the ensemble refinement of ubiquitin homodimer, a *C*_2_ non-crystallographic symmetry term was applied for each pair of interacting proteins.

For a protein complex, the structural refinement against CXMS restraints was first performed with a single-conformer (*N* = 1) representation for the complex. All the CXMS restraints could be satisfied for trypsin/BPTI and PP2Ac/IGBP1 complex. For EIN/HPr or ubiquitin/ubiquitin complexes, however, not all the cross-links could be accounted for. Thus we replicate the moving subunit to generate an *N* = 2, 3, 4, or 5 ensemble to represent the complex, and different conformers in the ensemble can overlap. Ambiguous distance restraints were employed: each restraint was applied to the C_α_ atom of Lys(*i*) of the fixed subunit and to the C_α_ atom of Lys(*j*) of any conformer of the moving subunit, in which *i* and *j* are the residue numbers of cross-linked lysine residues in Table 1. We defined the CXMS energy to be related to inverse sixth power of the distance between the C_α_ atoms of two cross-linked residues, and to be averaged over all conformers in the ensemble. As a result, the CXMS term has a steep dependence on distance and is biased towards the conformer with the shortest C_α_-C_α_ distance, which can be satisfied providing that one of the conformers in the ensemble has shorter-than-maximum lysine C_α_-C_α_ atom distance. The calculation was repeated 512 times starting from different random positions for each conformer of the moving subunit, and each calculation afforded a slightly different quaternary arrangement of the complex. Structures with no violations against CXMS restraints and no steric clashes were selected for further analysis. The flowchart for the ensemble refinement protocol against CXMS data was illustrated in Fig. S10.

The center-of-mass for one subunit with respect to the other subunit in the each CXMS model was calculated using an in-house Python script. The map projection with spherical coordinates was plotted using Gnuplot. The intermolecular NMR paramagnetic relaxation data were taken from previously published studies for EIN/HPr complex (Tang et al. 2006; Fawzi et al. 2010) and for ubiquitin homodimer (Liu et al. 2012), and ensemble refinement against the NMR data was performed as previously described. Reweighted atomic probability maps depicting the distribution of one subunit relative to another were calculated in Xplor-NIH (Schwieters et al. 2006) and were plotted at respective thresholds (Schwieters and Clore 2002). Structural figures were prepared with PyMOL (the PyMOL molecular graphics system).

## Electronic supplementary material

Below is the link to the electronic supplementary material.

Supplementary material 1 (pdf 6071 kb)

## Abbreviations

CXMS: Chemical cross-linking of proteins coupled with mass spectrometry analysis
NMR: Nuclear magnetic resonance
EM: Electron microscopy
BS^3^: Bis-sulfosuccinimidyl suberate
BS^2^G: Bis-sulfosuccinimidyl glutarate
PDH: Pimelic acid dihydrazide
BPTI: Bovine pancreatic trypsin inhibitor
PP2Ac: Phosphatase 2A catalytic subunit
IGBP1: Immunoglobulin binding protein 1
RMSD: Root-mean-square deviation
