# Supplementary material for: Visualizing the Ensemble Structures of Protein Complexes Using Chemical Cross-Linking Coupled with Mass Spectrometry
Source: Biophys Rep. 2015 Dec 28;1:127–38. doi: 10.1007/s41048-015-0015-y (PMC4871902; doi:10.1007/s41048-015-0015-y)
Supplement: Supplementary file 1 — Supplementary material 1 (pdf 6071 kb) [file 41048_2015_15_MOESM1_ESM.pdf]

Supplementary data

Visualizing the ensemble structures of protein complexes using chemical cross-linking coupled with mass spectrometry

Zhou Gong[1†], Yue-He Ding[2†], Xu Dong[1], Na Liu[2], E. Erquan Zhang[2], Meng-Qiu Dong[2*], Chun Tang[1*]

Table S1 Theoretical intermolecular cross-links based on PDB structure 2PTC

Cross-linked pair	Cα-Cα (Å)[a]
trypsin (60)- BPTI (46)	11.7
trypsin (145)- BPTI (15)	13.2
trypsin (224)- BPTI (15)	14.2
trypsin (60)- BPTI (15)	14.5
trypsin (188)- BPTI (15)	14.6
trypsin (156)- BPTI (15)	14.9
trypsin (230)- BPTI (15)	16.2
trypsin (222)- BPTI (15)	17.5
trypsin (159)- BPTI (15)	18.2
trypsin (60)- BPTI (41)	18.4
trypsin (169)- BPTI (15)	19.6
trypsin (87)- BPTI (46)	21.8
trypsin (107)- BPTI (15)	22.0
trypsin (204)- BPTI (15)	22.0
trypsin (87)- BPTI (15)	22.2
trypsin (224)- BPTI (41)	22.4
trypsin (145)- BPTI (41)	23.8

Table S2 Cross-validation for the structural refinement of PP2Ac-IGBP1 complex

Cross-link not used	Cα-Cα distance (Å) [a]	% satisfied [b]
PP2Ac(35)—IGBP1(163)	14.01±2.18	100%
PP2Ac(28)—IGBP1(158)	24.05±5.34	46%
PP2Ac(40)—IGBP1(166)	17.61±4.56	91.5%
PP2Ac(33)—IGBP1(166)	16.22±4.04	95.5%
PP2Ac(40)—IGBP1(163)	15.25±3.11	100%
PP2Ac(40)—IGBP1(158)	17.49±4.15	96.3%

[a] In the cross-validation, five out six CXMS restraints were used, and 128 complex structures were calculated. The Cα-Cα distance for the lysine pair of the unused cross-link was calculated and the averaged distance was reported

[b] The number of structures with back-calculated distance within the maximum length of the cross-linker (24 Å) relative to the total number of complex structures

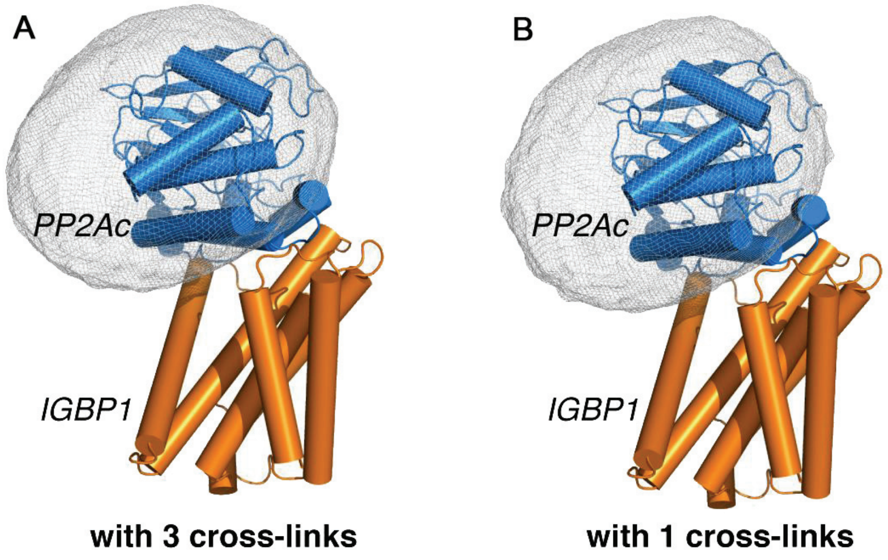

Fig. S1 Effect of the number of cross-links on the CXMS model. The structure for the complex between PP2AC and IGBP1 was calculated by refining against **A** three intermolecular cross-links, from PP2AC Lys^{40} to Lys^{158}, Lys^{163} or Lys^{166} in IGBP1, or **B** one intermolecular cross-links between PP2Ac Lys^{35} to IGBP1 Lys^{163}. The structures are superimposed by IGBP1 (orange cartoon), and the relative distribution of PPA2C in the CXMS models is shown as atomic probability map, plotted at 30% threshold and shown as gray meshes. For comparison, the crystal structure of the complex (PDB code 4IYP) is also superimposed, with PP2AC shown as blue cartoon

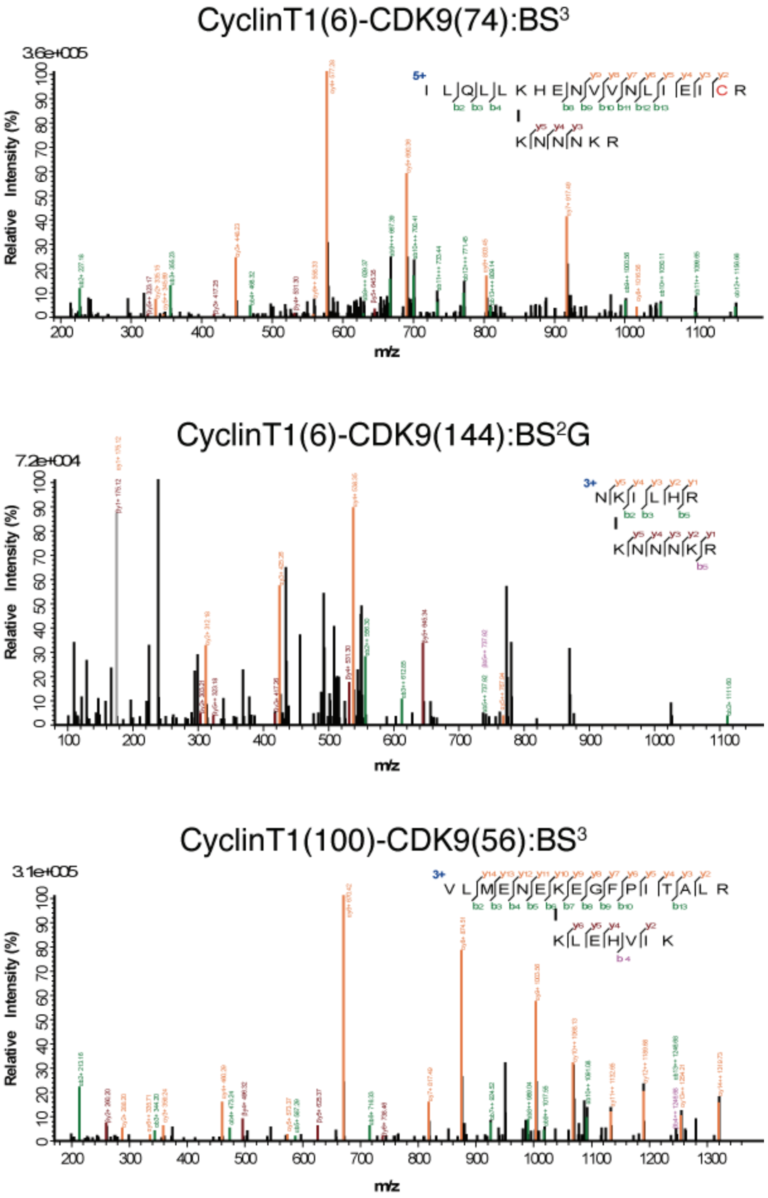

Fig. S2 MS2 spectra of cross-linked peptides identified from the BS^3- or BS^2G-treated, trypsin-digested CDK9/Cyclin-T1 complex. The peptide sequences are indicated

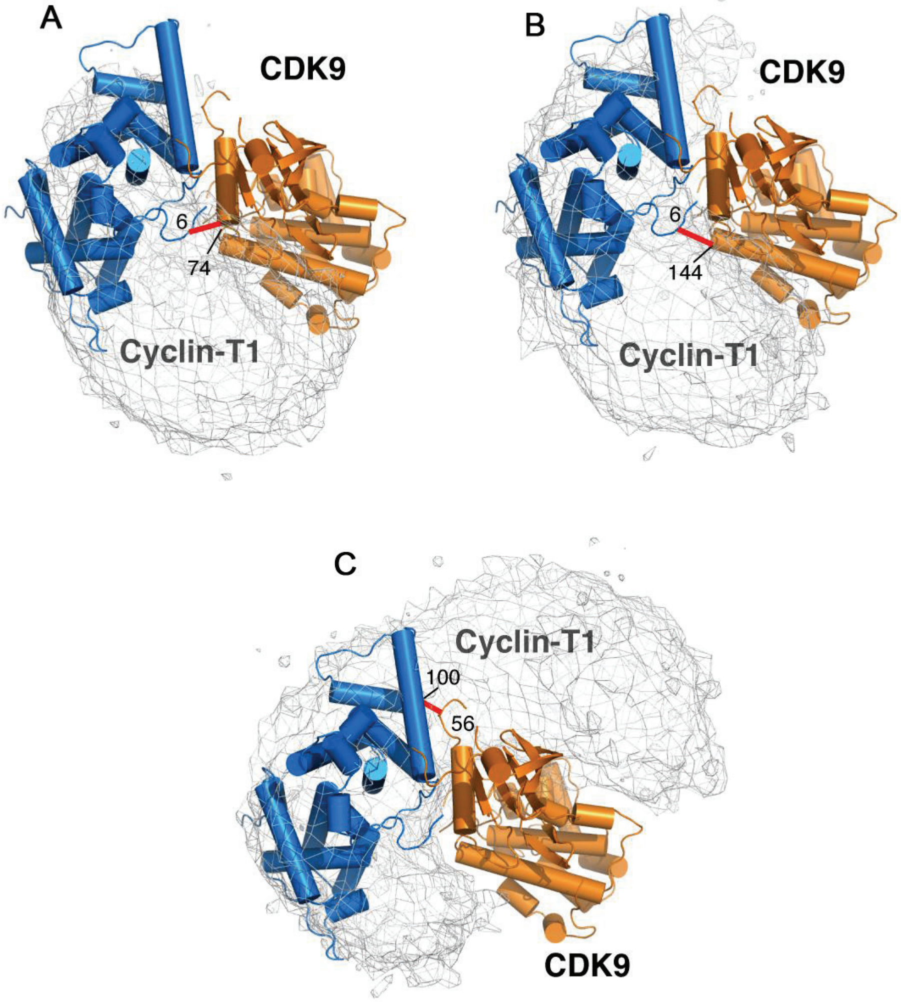

Fig. S3 Structural model for the CDK9/Cyclin-T1 complex refined against a single CXMS restraint. The cross-links are **A**Cyclin-T1 Lys^{6} to CDK9 Lys^{74}, **B**)Cyclin-T1 Lys^{6} to CDK9 Lys^{144}, **C** Cyclin-T1 Lys^{100} and CDK9 Lys^{56}. The CDK9 is superimposed for all the models (orange cartoon), and the structure of Cyclin-T1 is represented as an atomic probability map plotted at a 10% threshold (gray mesh). For comparison, the known complex structure is shown (PDB code 3BLH), with CDK9 superimposed and Cyclin-T1 shown as blue cartoon. Cross-linked residues are denoted with red bars

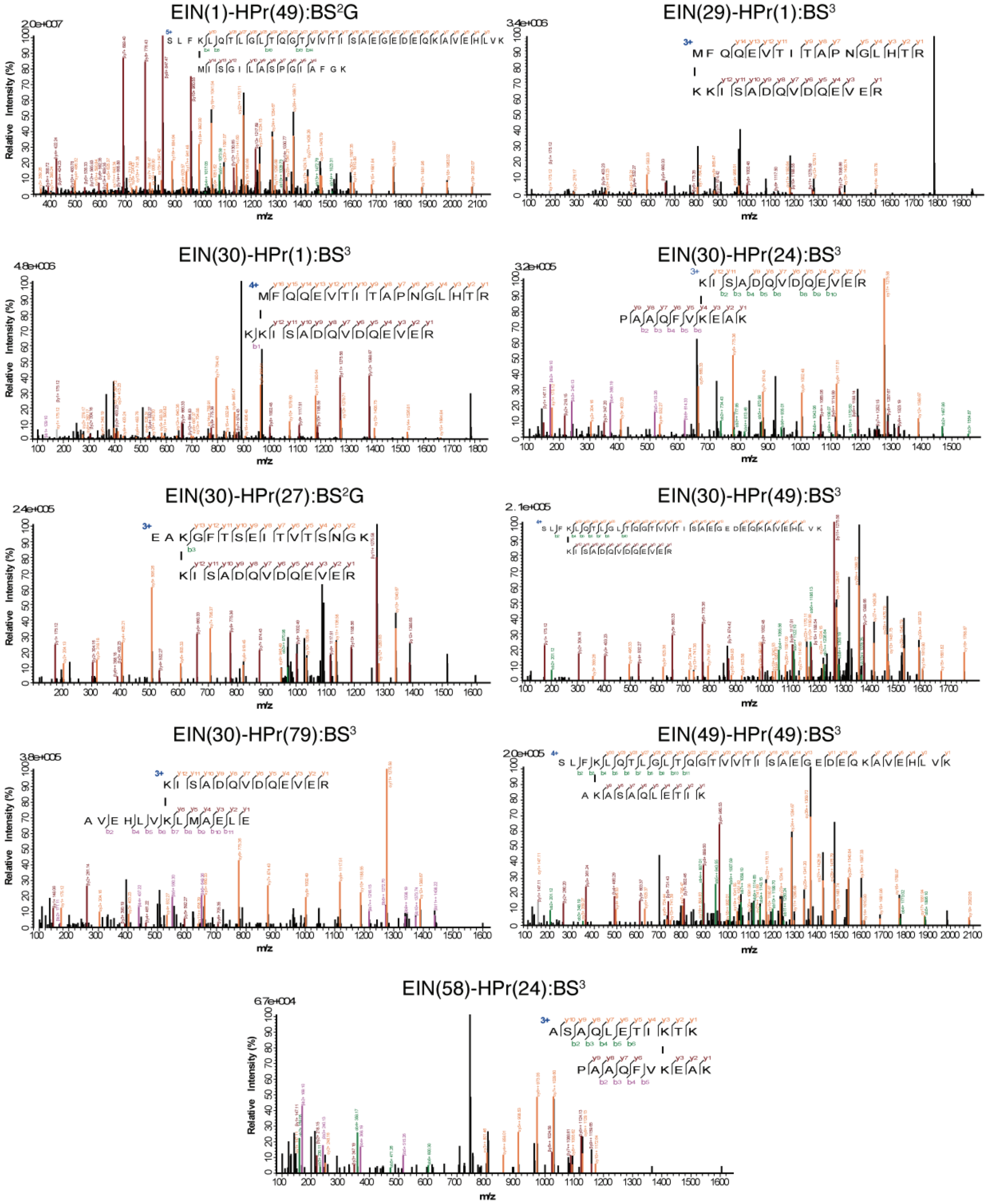

Fig. S4 MS2 spectra of cross-linked peptides for the transient EIN/HPr complex. Cross-linked with BS^3- or BS^2G, trypsin-digested, the identified peptide sequences are indicated. All the cross-links, except for the one between EIN Lys^{58} and HPr Lys^{24}, are not compatible with the known structure of the stereospecific complex (PDB code 3EZA)

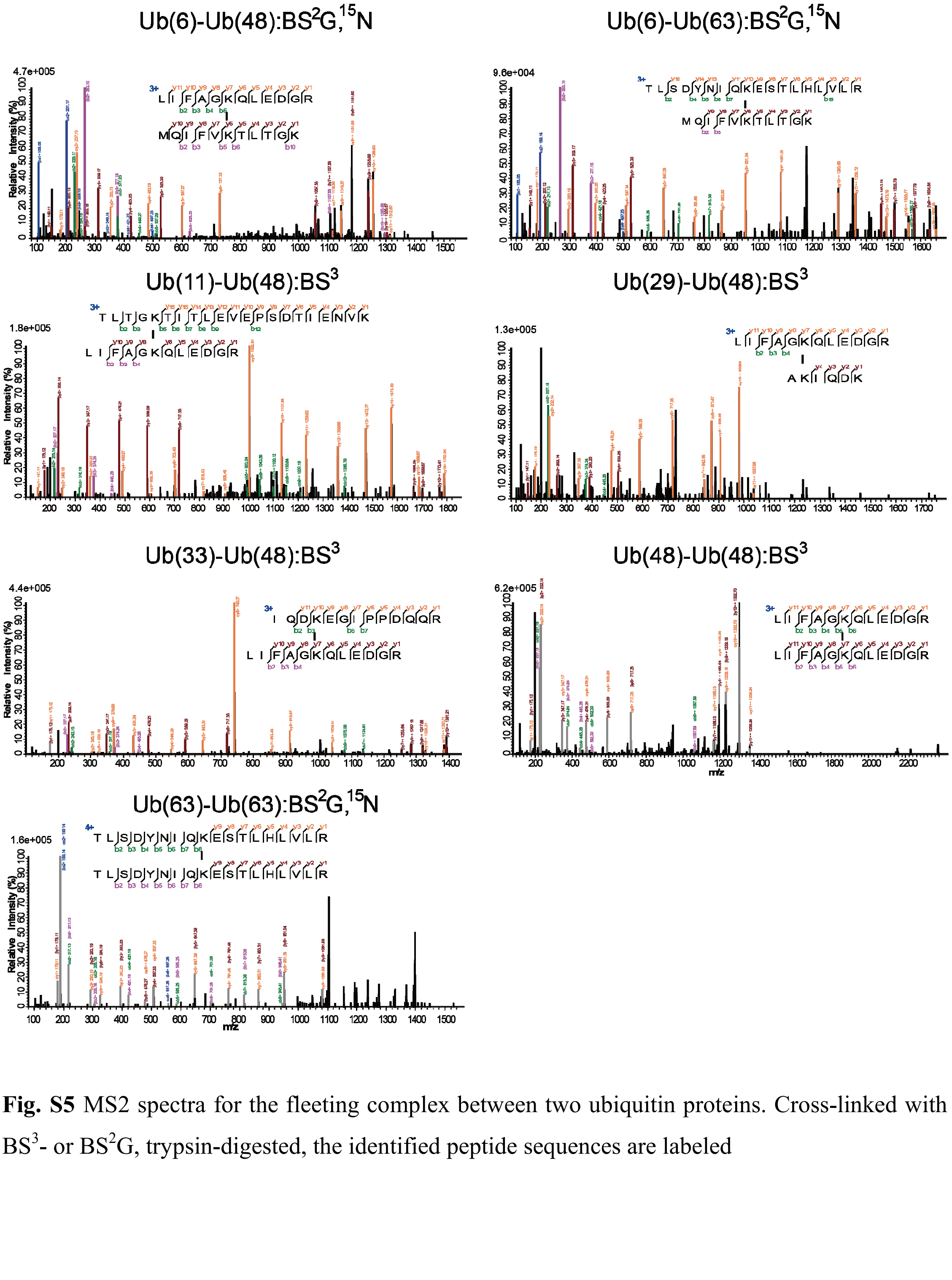

Fig. S5 MS2 spectra for the fleeting complex between two ubiquitin proteins. Cross-linked with BS^3- or BS^2G, trypsin-digested, the identified peptide sequences are labeled

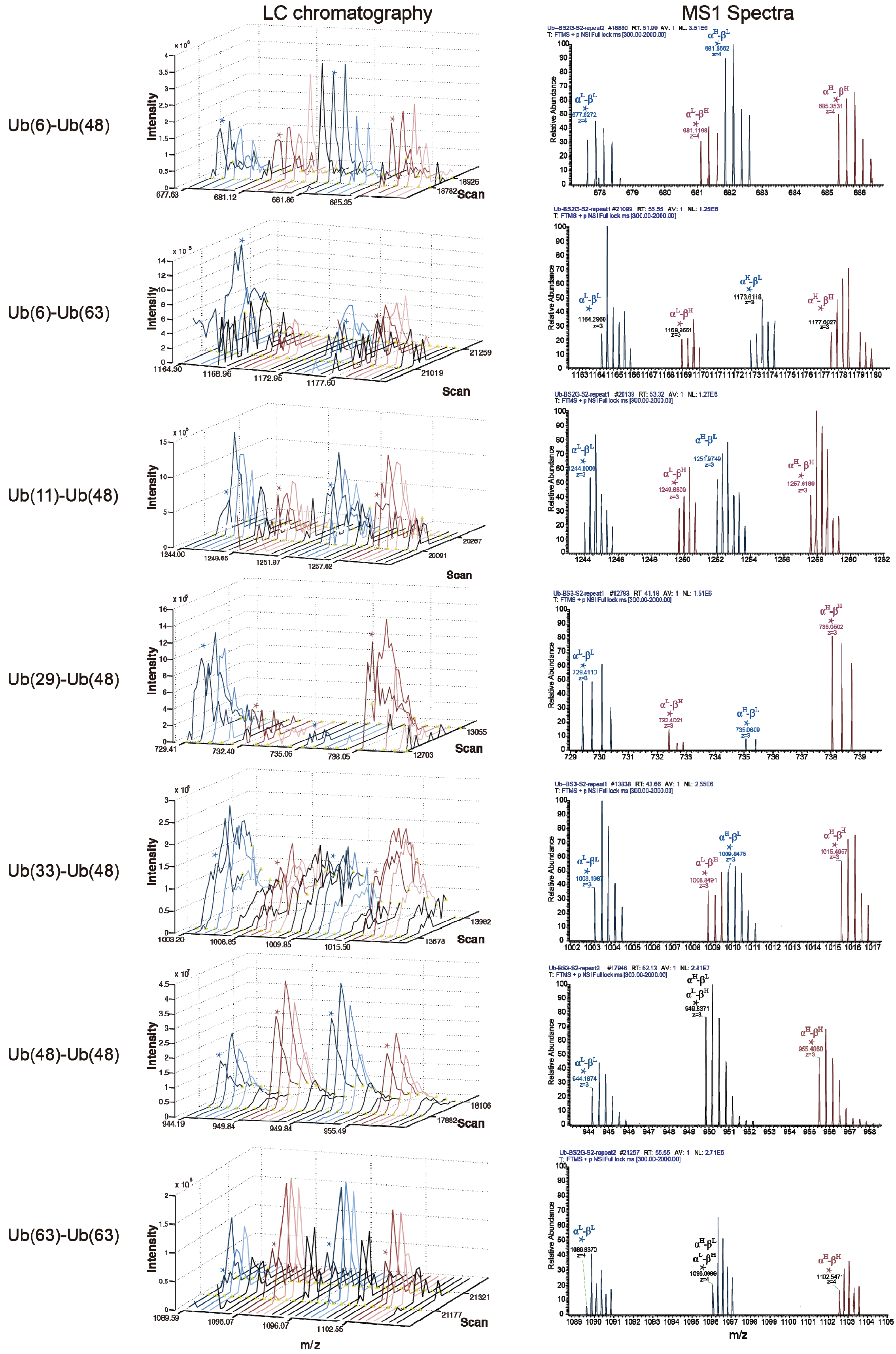
LC chromatography
MS1 Spectra
Ub(6)-Ub(48)
Ub(6)-Ub(63)
Ub(11)-Ub(48)
Ub(29)-Ub(48)
Ub(33)-Ub(48)
Ub(48)-Ub(48)
Ub(63)-Ub(63)
Intensity
Scan
m/z
Relative Abundance

Fig. S6 Identification of intermolecular cross-links for ubiquitin homodimer. Extracted ion chromatograms (XICs, left) and representative MS1 spectra (right) of the cross-linked peptides identified for an equimolar mixture of ^{14}N- and ^{15}N-labeled ubiquitin molecules. A cross-link identified between ^{14}N- and ^{15}N-labeled peptides manifests intermolecular interactions; a cross-link found only between two ^{14}N-labeled peptides or between two ^{15}N-labeled peptides manifests strictly intramolecular interactions. For each cross-link, the XICs consist of four clusters (α^L-β^L, α^L-β^H, α^H-β^L, and α^H-β^H), and each cluster starts with the XIC of the mono-isotopic peak (marked with * and the *m/z* value) followed by XICs of three additional isotopic peaks. The α^L and β^L denote ^{14}N-labeled peptides and α^H and β^H denote their ^{15}N-labeled counterparts. The α^L-β^H cluster and the α^H-β^L cluster may share one or more peaks; when the α-peptide is exactly the same as the β-peptide, such as in the case of Ub(48)-Ub(48), the two clusters completely overlap

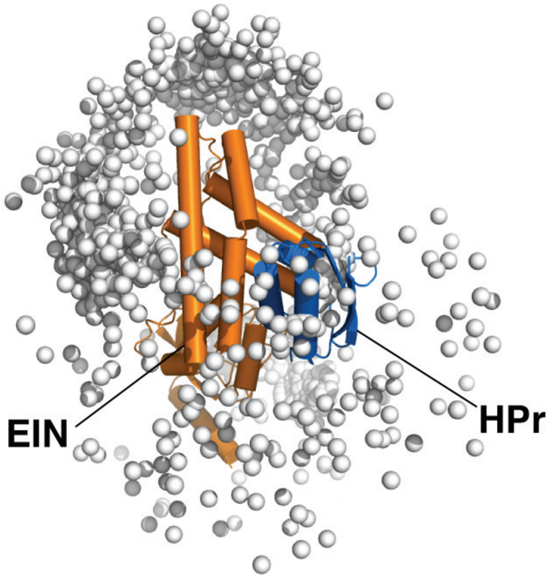

Fig. S7 Number of conformers in the ensemble required to satisfy CXMS data. Refinement of the EIN/HPr complex structure against CXMS distance restraints, with an $N = 5$ ensemble for the complex. The EIN is superimposed for all the models and is shown as an orange cartoon, and the center-of-mass for HPr in each CXMS model is represented as a gray sphere. A total of 307 five-conformer structures are plotted. For comparison, the specific complex structure is superimposed by EIN, with HPr shown as a blue cartoon

EIN(67)-HPr(85):PDH

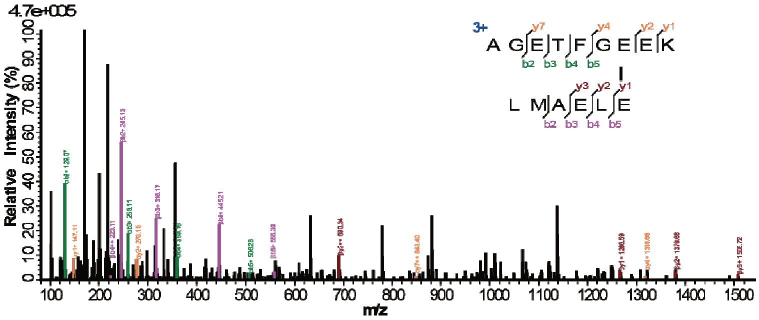

EIN(41)-HPr(85):PDH

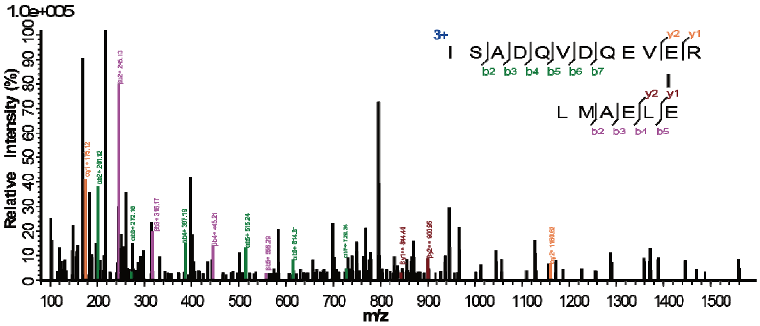

PDH cross-links	Spectral count	Best E-value	C_α-C_α (Å)
EIN(41)-HPr(85)	6	2.4E-09	41.2
EIN(67)-HPr(85)	33	3.6E-11	12.9

Fig. S8 Cross-links for EIN/HPr complex with PDH. Cross-linked peptides identified from the PDH-treated, trypsin-digested EIN/HPr complex. The peptide sequences in the MS2 spectra are indicated. Calculated from the known structure for the stereospecific complex (PDB code 3EZA), the Cα-Cα distances are 41.2 Å for the cross-links between EIN Glu^{41} and HPr Glu^{85}, and 12.9 Å between EIN Glu^{67} and HPr Glu^{85}

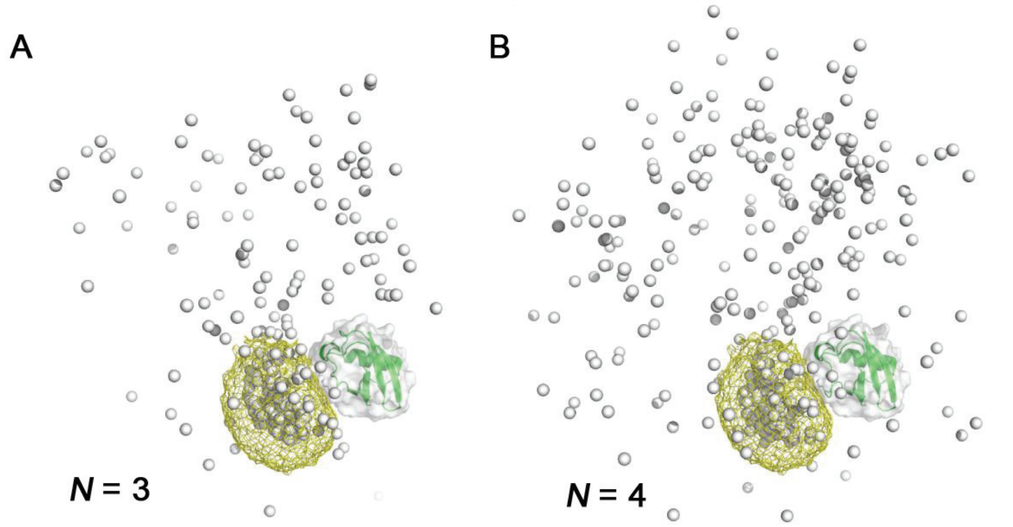

Fig. S9 Structural refinement of the ubiquitin homodimer. The refinement was performed with **A** $N = 3$ ensemble and **B** $N = 4$ ensemble for the dimer. One of the subunits is superimposed (green cartoon and gray surface) and the center-of-mass of the other subunit is represented as a gray sphere. A total of 114 three-conformer structures (342 conformers) and 115 four-conformer structures (460 conformers) were plotted in **A** and **B**, respectively. For comparison, probabilistic distribution of ubiquitin dimer using a two-conformer representation was shown (plotted at 15% threshold, yellow mesh). Since the two-conformer representation is sufficient to account for the CXMS data, the extra conformers in the three- and four-conformer ensembles scatter around and are not clustered

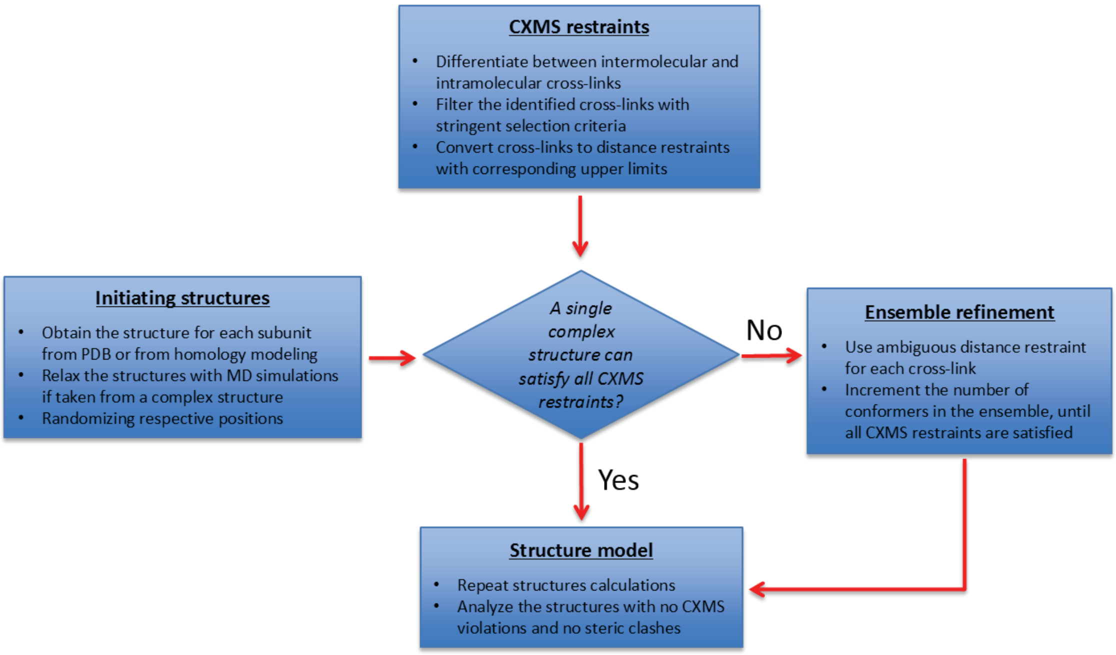

Fig. S10 Flowchart for ensemble refinement procedure against CXMS distance restraints
